# Supplementary material for: The effects of genital myiasis on the diversity of the vaginal microbiota in female Bactrian camels
Source: BMC Vet Res. 2022 Mar 5;18:87. doi: 10.1186/s12917-022-03189-5 (PMC8897907; doi:10.1186/s12917-022-03189-5)
Supplement: Supplementary file 5 — Additional file 5. [file 12917_2022_3189_MOESM5_ESM.zip › MPL201709200_16s_yy/Treat1/css/js_css/jsPDF-master.zip!/jsPDF-master/examples/html2pdf/showcase.html]

Html2Pdf


# Html2Pdf

This demo uses Html2Canvas.js to render HTML.   
Instead of using an HTML canvas however, a canvas wrapper using jsPDF is substituted. The *context2d* provided by the wrapper calls native PDF rendering methods.

A PDF of this page will be inserted into the right margin.

## Colors

red rgb(0,255,0) rgba(0,0,0,.5) #0000FF #0FF

## Text Alignment

left

center

right

## Margins and Padding

Red

Green

Blue

## Borders

Single

---

Double

## Font Style

Normal

Italic

Oblique

## Lists

- apples
- oranges
- pears
- peaches
- lemons
- limes

## Font Size

10px

20px

30px

20pt

1em

2em
